# Transmission Efficiency of a MEMS Laser Fuze for Safety and Arming

**DOI:** 10.3390/mi16121345

**Published:** 2025-11-28

**Authors:** Kuang Fang, Shanglong Xu, Wenzhi Qin, Jiangnan Ran, Chao Chen, Peng Yang, Yalong Dai

**Affiliations:** 1 Yangtze Delta Region Institute (Huzhou), University of Electronic Science and Technology of China, Huzhou 313001, China; kuang.fang@outlook.com (K.F.);; 2 National Key Laboratory of Integrated Circuits and Microsystems, Chongqing 400000, China; 3 Institute of Chemical Material, China Academy of Engineering Physics, Mianyang 621900, China

**Keywords:** laser fuze, MEMS, safety and arming device, coupling efficiency

## Abstract

Owing to their superior performance in countering electromagnetic interference on the battlefield, laser fuzes have become a promising candidate for application in munition systems. However, as the short-pulse laser is activated by an electrical signal, the possibility of accidental emissions caused by logic device failure cannot be ruled out, making it vulnerable under the effects of strong electromagnetic coupling. Integrating an encrypted, MEMS-based Safety and Arming Device (SAD) into the energy channel to control the propagation of short-pulse lasers can significantly enhance the safety level of munition systems. In the present work, the effect of MEMS SAD integration on laser propagation is investigated. The results demonstrate that the insertion of a MEMS SAD does not introduce significant attenuation of short-pulse laser propagation. A firing test is conducted using the laser-driven flyer detonator to verify the safety, charging mechanism, and function to provide a comprehensive characterization of the laser fuze. To guarantee the initiation of insensitive explosives, the coupling efficiency and laser transmission energy density of multi-mode quartz fibers are studied.

## 1. Introduction

Laser fuze is an important field of laser ranging applications, and the emergence of laser fuzes greatly improves combat efficiency [1,2]. All laser units are still ultimately controlled and activated by electrical systems, which contain a large number of logic devices [3,4]. Device miniaturization provides additional space within the weapon for sensors and other electronic equipment [5]. But MEMS (Micro-Electro-Mechanical System)-based fuzes are also affected by external interference, such as strong light, smoke, multi-path, and interference pulses emitted by hostile forces [5,6]. Under strong magnetic field interference, potential safety hazards for any logic devices exist, which might directly lead to the emission of accidental detonation energy [7,8]. Therefore, integrating a switch sensor on the energy transfer channel is necessary to ensure the safety of laser fuzes [9].

Currently, researchers are working to apply laser fuzes to more weapon systems and continuously improve their performance [10,11,12]. They are studying the response and response time of laser fuzes to improve their accuracy and rapid response ability [13,14]. These studies typically require the use of high-speed cameras to record the interaction between lasers and explosives, as well as developing more sensitive detection devices to measure reaction time. They are also studying the physical characteristics of laser sources to improve their stability [15,16]. This involves exploring new materials and manufacturing methods to improve the efficiency of lasers and reduce their impact on the environment [17]. In addition, the controllability and safety of laser fuzes are also being investigated [18,19]. These studies mainly focus on designing more sophisticated control systems to ensure that the conditions for activating laser fuzes are met. It is necessary to ensure the safety and stability of the fuze system to avoid misoperation and malfunctions. Finally, the anti-interference performance of laser fuzes is an important research field [20,21,22]. It mainly involves testing and evaluating the performance of laser fuzes in complex environments, such as adverse weather, electromagnetic interference, and others. Guo Z. et al. [23] established a virtual simulation model for FMCW laser transmission using a unified professional particle system. The simulation results indicated that the factors affecting the signal-to-noise ratio of the echo signal were related to the interference of smoke and the performance changes of detection. The shielding performance of the integrated shielding model studied by Dong G. et al. [24] can ensure the normal operation of the laser fuzes in strong electromagnetic interference environments, with the electromagnetic shielding performance reaching 40 dB in the 0–8 GHz frequency band.

The high-speed movement of the carrier platform will cause disturbances in the surrounding air, which leads to uneven changes in the refractive index of the air, ultimately affecting the propagation process of the laser [25]. This also causes problems such as changes in the optical path of the imaging beam, spot shift, and energy attenuation, which in turn affect the detection performance of the laser [26]. A fuze laser combination system was proposed for a single projectile based on photoelectric conversion technology and optical communication technology. It is expected to have high setting accuracy, stable data transmission, and strong anti-interference ability. However, a MEMS fuze system is novel and has many advantages. R.A. Lake [27] used the electro-thermal principle to actuate an SA device and successfully minimized its size into millimeters by utilizing surface micromachining technology. H. Pezous [28] reported the integration of a MEMS SA and firing device, and the total size (control circuit included) was less than 10 mm × 10 mm × 10 mm. Hu et al. [29] designed, manufactured, and tested new multi-layer stacking equipment (MEMS fuze). MEMS technology is used in the weapon system. But they did not study the effect of MEMS encryption devices on laser transmission energy density and coupling efficiency.

In this work, a fiber-coupled laser fuze is presented with one end coupled to a MEMS SAD and the other end coupled to a laser-driven flyer detonator. To provide a comprehensive characterization of the system, the laser energy coupling efficiency is investigated to verify the effect of the MEMS SAD on short-pulse laser propagation. A firing test with a laser-driven flyer detonator is conducted as a functional manifestation of the integrated system. The coupling ability of optical fibers is studied to ensure sufficient energy for the initiation of insensitive ammunition.

## 2. System Configuration and Test

### 2.1. System Configuration

The laser fuze system consists of a Nd:YAG laser source with a flat-top distribution of laser energy, a fundamental wavelength of 1064 nm, and a pulse width of 7 ns. The short-pulse laser emitted by the source is modulated by a group of optical lenses and focused onto the input face of the optical fiber via the channel on the MEMS SAD, with the on–off status controlled by an interrupter, which is driven by an encrypted signal. The other end of the fiber, coupled with a laser-driven flyer detonator, is placed in an explosion-proof tank for safety reasons.

**Figure 1:**
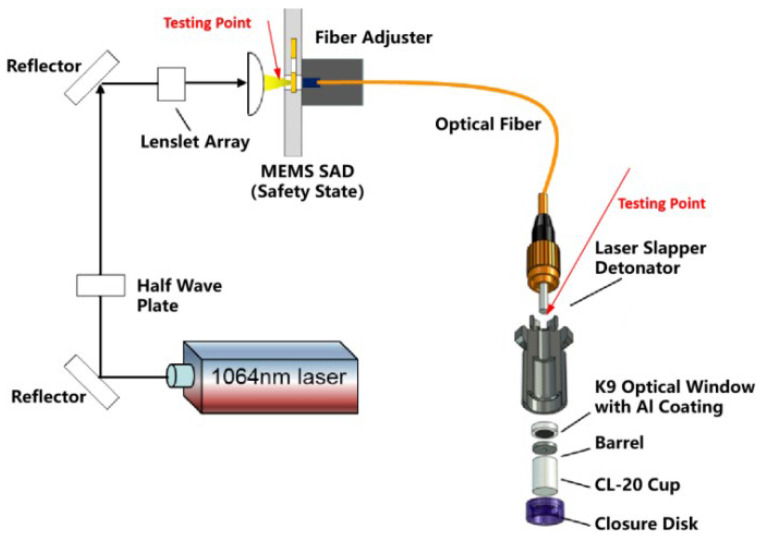
MEMS-Based Laser Fuze and Laser Attenuation Test.

Figure 1 illustrates the laser-driven flyer detonator utilized in the present work. The detonator consists of an optical window coated with an aluminum foil with a thickness of 20 μm, a steel barrel for shearing of the aluminum foil, an explosive cup, and the package shell. The short-pulse energy transfer through the optical fiber will hit the optical window (K9 glass, Φ5 mm × 1 mm) and then the aluminum foil will be activated by the laser and simultaneously shared by the steel barrel, resulting in a high speed flyer, which finally collides with the explosive cup for initiation. As for the MEMS SAD, its interrupter is driven by a signal, with timing and voltage level encryption, to slide on the silicon substrate with a range of more than 1 mm, forming a switch to control the propagation of the short-pulse laser.

To validate the compatibility of MEMS SAD integration, the energy level of a short pulse laser at the input of the MEMS SAD and the output of the optical fiber were examined. According to the test results, there was no significant laser attenuation caused by the insertion of the MEMS SAD.

The laser source employed was a single-pulse, Q-switched Nd:YAG laser operating at the fundamental wavelength of 1064 nm. The laser generated pulses with a full width at half maximum (FWHM) of 7.6 ns. The pulse temporal profile was nearly Gaussian, with a typical rise time of approximately 5 ns and a decay time of approximately 6 ns. The energy transmission medium was a multimode quartz silica fiber with a numerical aperture (NA) of 0.22. The fiber core diameters used in the experiments were 0.6 mm and 0.8 mm, as specified in the text.

### 2.2. MEMS SAD

To meet the safety standards of fuze applications, the MEMS SAD in this work incorporates advantages such as a large travel range (for interrupters), resetability, and encryptability. The core innovation lies in a mechanical coding system that enhances anti-electromagnetic interference by utilizing codes not found in nature, thereby eliminating backdoors associated with electronic logic devices. A hybrid fabrication process is employed to improve the reliability of laser interruption. The device dimensions are 14 mm in length, 10.5 mm in width, and 753 μm in thickness, excluding bonding adhesive.

**Figure 2:**
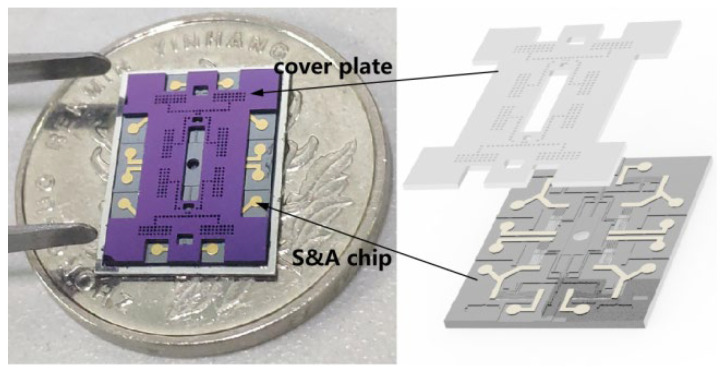
Components of the MEMS SAD.

**Figure 3:**
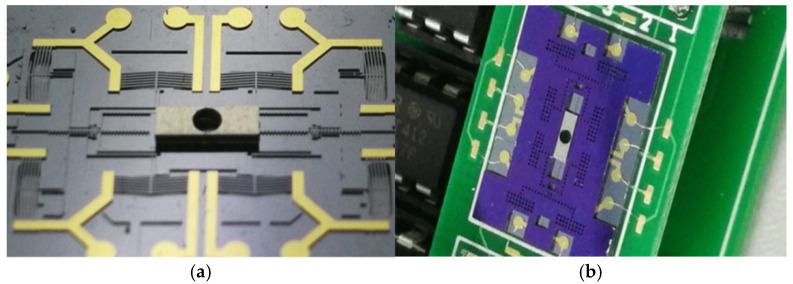
Ni plate electroplated (**a**) on the silicon S&A chip (**b**) the packaged MEMS SAD.

The MEMS SAD comprises a silicon cover plate and an S&A (safety and arming) chip, as illustrated in Figure 2. The cover plate acts as a protective mask, while the S&A chip—fabricated on a Silicon-On-Insulator (SOI) wafer—serves as the core unit for interrupter actuation. The chip features a slider mechanism with interlocking teeth and pawls, driven by electrothermal actuators. To enhance the structural integrity of the silicon interrupter (fabricated on the 50 μm thick device layer of the SOI wafer), an electroplating process forms a 300 μm thick nickel plate on the interrupter, as shown in Figure 3a. The packaged MEMS SAD is depicted in Figure 3b.

**Figure 4:**
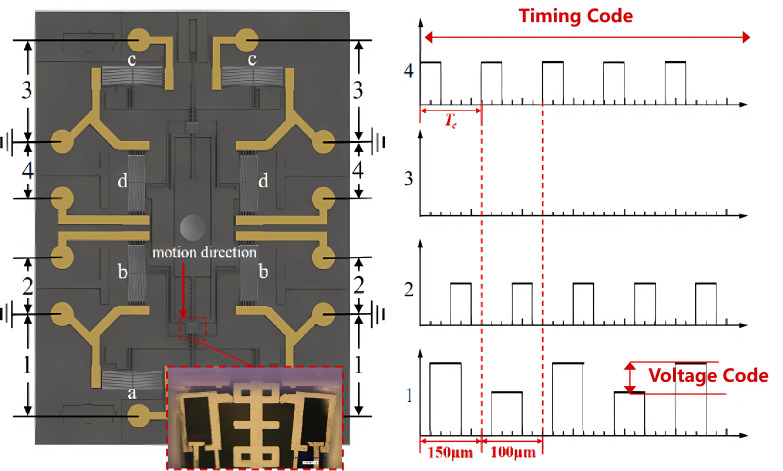
Coding method of the MEMS SAD.

The driving principle is based on the electrothermal effect, consistent with prior work [30]. It utilizes eight electrothermal actuators that coordinate to accumulate incremental displacements. Each actuator generates thermal expansion, pushing against a slider with pawl-and-tooth engagements, resulting in a millimeter-scale travel range for the interrupter. Specifically, the actuators are paired into four sets, with each set controlling directional movement through timed signals. The encryption mechanism involves two-level coding, timing code and voltage code, as detailed in Figure 4. The timing code defines the signal sequence on the time axis for the four actuator pairs, ensuring synchronization. The voltage code specifies voltage variations between 13.5 V and 14.8 V for the actuator pair responsible for slider movement, aligning the pawl with gaps on the slider. If either code is unmet, the pawl fails to engage, disabling arming or disarming processes. This approach ensures high security by leveraging unique, non-natural code sequences.

### 2.3. System Compatibility and Test

Switch tests validated the laser propagation interruption capability of the integrated MEMS SAD. Two emission tests using a laser-driven flyer detonator assessed the system’s initiation ability and configuration repeatability. Laser energy measurements at the input and output ends confirmed compatibility, with no significant attenuation observed due to MEMS SAD insertion.

**Figure 5:**
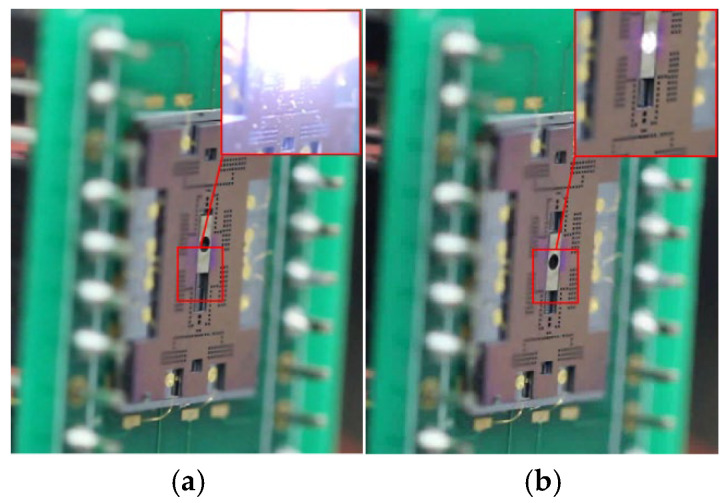
Switching test of the MEMS SAD: (**a**) the safety state and screenshot when the laser is intercepted; (**b**) the arming state and screenshot when the laser is passing through the MEMS SAD.

In the switch test, an encrypted signal sequence controls the interrupter’s transition between safe and armed states (travel range ~1 mm). In the safe state, the interrupter blocks the central hole of the MEMS SAD substrate, intercepting the laser with the 300 μm thick nickel plate, as shown in Figure 5a. In the armed state, hole alignment opens the laser channel, allowing the short-pulse laser to pass through and initiate the laser-driven flyer detonator via optical fiber coupling, as captured in Figure 5b. Both detonator tests successfully initiated, demonstrating reliable functionality.

The results highlight the system’s integration efficacy, focusing on performance metrics such as initiation success and energy transmission consistency, without delving into MEMS SAD internals.

## 3. Laser Coupling Efficiency

The initiation of high-energy insensitive munitions through a high-energy-density laser is of great interest, whilst also being a great challenge. Specifically, when a high-energy-density laser is coupled to an optical fiber, the input face or the internal part of the fiber will most likely be damaged by laser-induced interaction [2,3]. As a result, it is necessary to have a better understanding of the coupling efficiency and maximal laser transmission energy density of the optical fiber [4,5].

**Figure 6:**
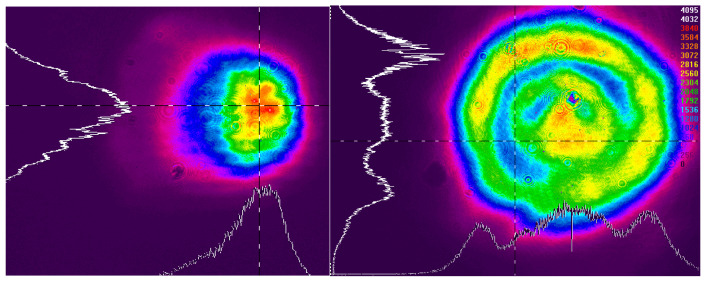
Gaussian distribution (**left**) and flat-top distribution (**right**).

The laser coupling experiments were performed using a single Nd:YAG laser source (Lumispot, Suzhou, China, wavelength: 1064 nm, pulse width: 7.6 ns), which was capable of operating in two modes: one producing a fundamental Gaussian beam profile and the other incorporating an integrated beam-shaping optical element to generate a flat-top (top-hat) profile, as shown in Figure 6. This approach ensured that critical laser parameters such as wavelength, pulse duration, and coherence were identical for both profiles, isolating the energy distribution as the primary variable under investigation.

The output beam from the laser was focused onto the input face of the optical fiber using a 300 mm focal length lens. The beam diameter at the fiber input face was carefully adjusted by controlling the lens position. For both beam profiles, the focusing condition was set such that the $\frac{1}{e^{2}}$ diameter of the Gaussian beam and the top-hat diameter of the flat-top beam each covered approximately 80% of the fiber core diameter. This standardization maximized coupling efficiency while minimizing the risk of clipping the fiber cladding, ensuring a consistent and equitable comparison between the two profiles. For example, the target spot diameter was approximately 0.48 mm for the 0.6 mm core fiber.

**Figure 7:**
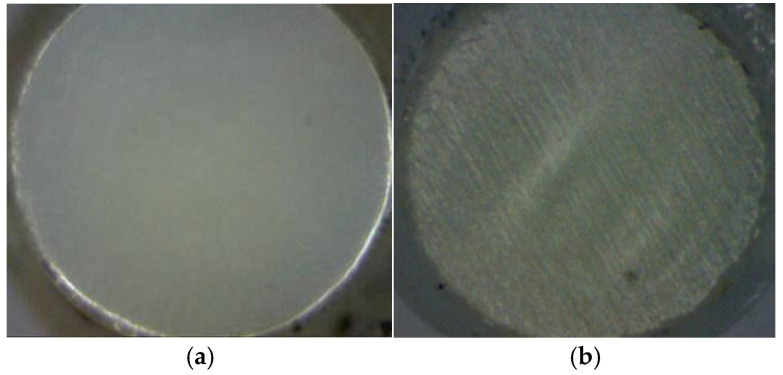
Surface profiles of polished incident faces. (**a**) Ra = 0.08 μm; (**b**) Ra = 0.25 μm.

The influence of fiber surface roughness on laser coupling performance was further studied by using the coupling system. Multimode quartz fibers with 0.6 mm and 0.8 mm core diameters were used. Optical fibers with different surface roughness were obtained by grinding their incident faces using grinding papers. Specifically, the incident face of optical fibers was polished using 12,500-screen polishing paper and 2000-screen abrasive paper. Accordingly, the roughness of the fiber surface was 0.08 μm and 0.25 μm. Figure 7 shows the surface profiles of the polished incident face of the optical fibers. The tail ends of both fibers were polished using 12,500-screen polishing paper.

### 3.1. Results of Gaussian Distribution

The coupling efficiency was characterized by applying a series of single laser pulses to the fiber input. The energy of each pulse was gradually incremented until a significant drop in coupling efficiency indicated fiber damage. Each data point in the subsequent data analysis chart represents a single, independent laser shot on a fresh spot of the fiber sample (or until damage occurred). A sufficiently long time interval (several seconds) was maintained between consecutive pulses to ensure that each test was an independent event, free from thermal accumulation effects.

Gradually increasing laser energy was coupled to the polished incident faces (roughness 0.08 μm) until the coupling efficiency started to drop dramatically. The transmitted laser energy was detected at the tail ends using an Ophir energy meter. The point at which the coupling efficiency begins to drop precipitously marks the damage threshold of the optical fiber. After that, the coupling efficiency drops dramatically, reflecting the failure of the optical fiber.

**Figure 8:**
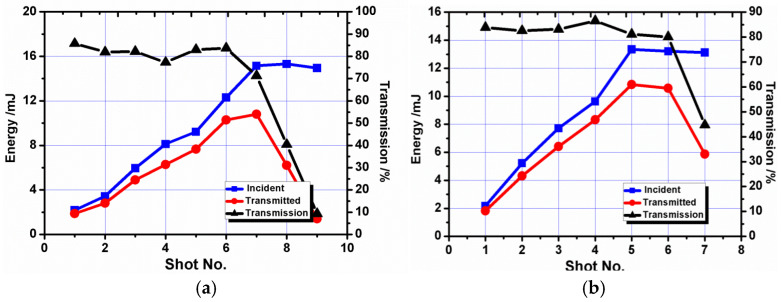
Coupling threshold test results for fibers with a surface roughness of 0.08 μm. (**a**) Optical Fiber 1 (OF1) with a 0.6 mm core diameter; (**b**) Optical Fiber 2 (OF2) with a 0.8 mm core diameter.

As shown in Figure 8, the transmission efficiency of OF1 is generally in the range of 79% to 84%. When the energy increased to 15.5 mJ, the maximum energy detected at the fiber tail end was 10.2 mJ, and its corresponding energy density was ~3.6 J/cm^2^. Another two shots were fired onto the fiber at 15 mJ, and the output energy was reduced to 5 mJ and 2 mJ, respectively. Their corresponding coupling efficiencies are 40% and 10%. The result is almost the same as OF2. The maximum transmission energy is 10.8 mJ, and its corresponding energy density is ~2.1 J/cm^2^. The transmission efficiency is in the range of 81% to 85%.

**Figure 9:**
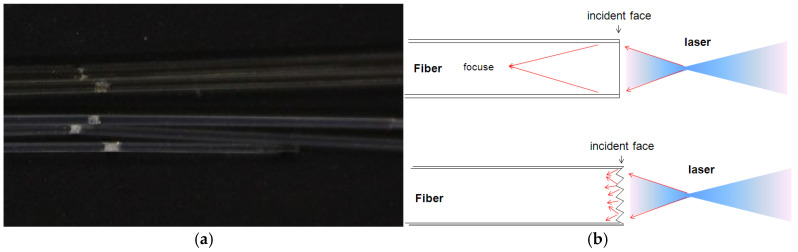
Damage inside the fiber (**a**) and optical path diagram of the fiber surface (**b**).

After the tests, severe damage was observed inside the fiber (Figure 9a), indicating irreversible damage to the optical fiber. It is considered that when the laser enters the fiber, the smooth surface causes the laser to focus inside the optical fiber and form a high-energy-density spot, which leads to internal damage. Thus, abrasive paper with a roughness of 2000 screen was used to grind the input face, increasing the surface roughness to 0.25 μm. Irregular scattering incidence is more likely to occur, and the possibility of laser beam focusing inside the fiber will be greatly reduced (Figure 9b).

**Figure 10:**
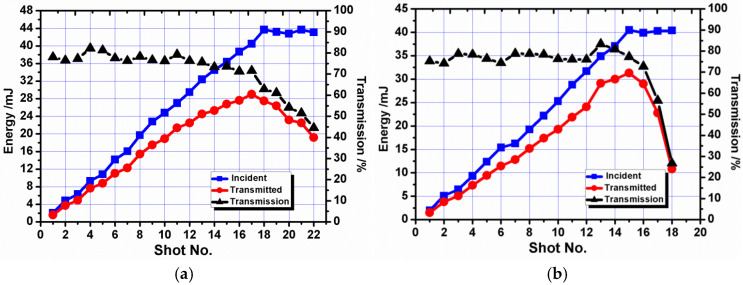
Coupling threshold test results for fibers with a surface roughness of 0.25 μm. (**a**) Optical Fiber 3 (OF3) with a 0.6 mm core diameter; (**b**) Optical Fiber 4 (OF4) with a 0.8 mm core diameter.

Figure 10 provides the results of optical fibers with diameters of 0.6 mm and 0.8 mm and a surface roughness of 0.25 μm. The transmission efficiency of OF3 is generally in the range of 74% to 81%, lower than OF1. The maximum transmitted energy is 30.2 mJ, 20 mJ more than OF1. Similarly, the transmission efficiency of OF4 is generally in the range of 77% to 81%, lower than OF2. The maximum transmitted energy is 52.1 mJ, 41.3 mJ more than OF2. The fibers with high roughness obtain higher transmission energy but lower transmission efficiency.

**Figure 11:**
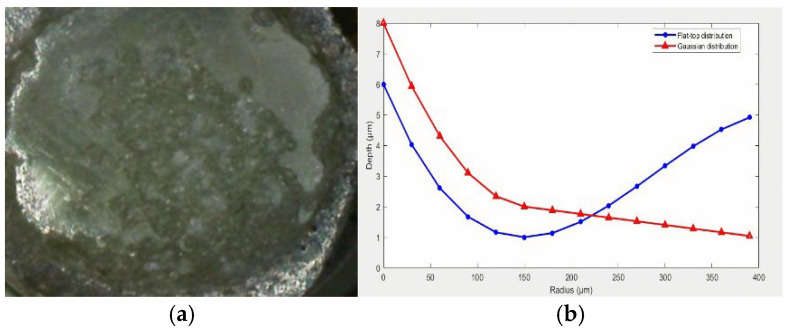
(**a**) Multiple sites of minor damage on the input face of the optical fiber. (**b**) Typical pit-like damage occurring near the core axis.

After testing, no visible damage was observed inside the optical fiber, but multiple sites of minor damage were found on the input surface, as shown in Figure 11a. This indicates that the laser is not focused inside the fiber. Instead, the incident surface is ablated. When the laser power density is about 3 GW/cm^2^, the depth of the damage pit is 0.8 μm. The high peak power density at the center of the light spot increases the damage to the fiber end face for a Gaussian distribution of the laser beam. The Gaussian distribution of power is

$$E(r)=E_{0}e^{-2r^{2}/w_{0}^{2}}$$

Total energy of the laser is

$$Q=\int_{0}^{2\pi }\int_{0}^{\infty }E_{0}e^{-2r^{2}/w_{0}^{2}}rdrd\theta =\pi w_{0}^{2}E_{0}/2$$

$w_{0}$ is the spot radius and E is the spot energy density.

The laser power density for typical pit-like damage occurring near the core axis is around 2–4 GW/cm^2^, with an area of 6 µm and a depth of 0.8 µm (Figure 11b). The power density or energy density at the center of the spot is 2–6 times higher than the average value, so the high peak power density at the center of the spot increases the possibility of fiber end face damage.

### 3.2. Results of “Flat-Top” Distribution

**Figure 12:**
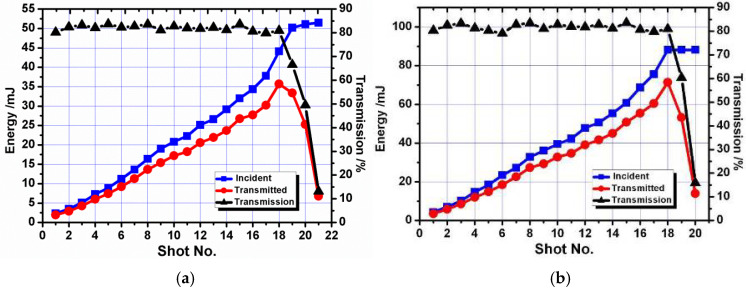
Coupling threshold test results for fibers with a surface roughness of 0.08 μm. (**a**) Optical Fiber 5 (OF5) with a 0.6 mm core diameter; (**b**) Optical Fiber 6 (OF6) with a 0.8 mm core diameter.

**Figure 13:**
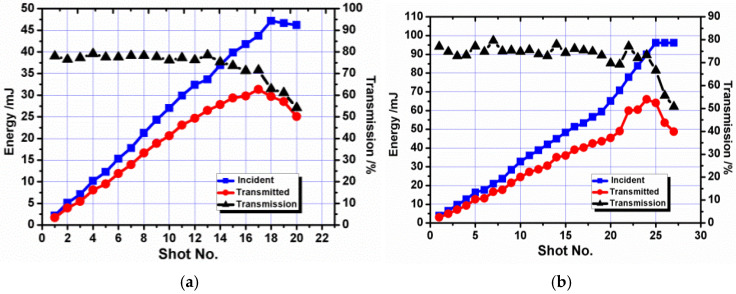
Coupling threshold test results for fibers with a surface roughness of 0.25 μm. (**a**) Optical Fiber 5 (OF7) with a 0.6 mm core diameter; (**b**) Optical Fiber 6 (OF8) with a 0.8 mm core diameter.

The same test process was conducted by utilizing the laser beam of flat-top distribution. Figure 12 illustrates the experimental results of the laser coupling test for optical fibers with diameters of 0.6 mm and 0.8 mm, and the roughness of the input surfaces is 0.08 μm for both. The transmission efficiency of OF5 is generally in the range of 80% to 83%. When the energy increased to 50.3 mJ, the maximum energy detected at the fiber tail end was 35.7 mJ, and its corresponding energy density was ~12.6 J/cm^2^. For the fiber with a 0.8 mm core diameter, the transmission efficiency was in the range of 80% to 84%, and the maximum transmitted energy was 71.4 mJ. Optical fibers with a surface roughness of 0.25 μm were utilized to complete the test in the same way. Figure 13 provides the coupling test results of optical fibers with diameters of 0.6 mm and 0.8 mm. The transmission efficiency of OF7 is generally in the range of 72% to 79%. When the energy increased to 47.4 mJ, the maximum energy detected at the fiber tail end was 31.4 mJ, and its corresponding energy density was ~11 J/cm^2^. For OF8, the maximum energy detected at the fiber tail was 64.3 mJ, and the transmission efficiency was generally in the range of 71% to 78%.

Comparing the above results, it is found that for Gaussian distributed laser beams, fibers with low input surface roughness can cause the laser to focus inside the fiber, forming high-energy-density light spots, leading to serious damage. Thus, it is difficult to obtain high transmission energy. Conversely, a rough input face suppresses internal focusing by promoting diffuse scattering of the incident laser light. Damage to the incident surface results in lower transmission efficiency but higher transmission energy. For a flat-top distribution, due to the uniform distribution of laser energy, it is less likely to form a high-energy-density spot compared to the Gaussian distribution in the center, thus achieving higher transmission energy. In general, the MEMS laser fuzes we designed have higher conversion efficiency compared to conventional sizes (below 65%) for these cases.

In addition, different from the situation of a Gaussian distribution, the high energy density spot would not be the main concern for a flat-top distribution because of its uniform laser energy distribution. Therefore, increasing the roughness of the incident surface will not have a significant positive effect. On the contrary, irregular scattering incidence will reduce transmission efficiency and maximum transmission energy.

## 4. Conclusions

In the present work, a MEMS-based laser fuze is proposed, with compact dimensions of 14 mm × 10.5 mm × 753 μm (excluding bonding adhesive). To counter electromagnetic interference on the battlefield, the MEMS SAD incorporates encryption mechanisms using timing and voltage codes, enabling a large stroke range through accumulated electrothermal actuation. This ensures reliable opening and closing of the laser propagation channel for optical fibers with diameters of 0.6 mm and 0.8 mm. Compatibility tests between the MEMS SAD and laser detonation systems confirm no significant propagation attenuation, demonstrating effective integration for safe arming applications.

The experimental investigation on laser energy transmission reveals critical influence mechanisms of energy distribution and fiber input surface roughness on transmission efficiency. For Gaussian-distributed lasers, the energy concentration at the center results in high peak power density. With low surface roughness (0.08 μm), the smooth input face causes laser focusing inside the fiber, leading to internal damage and limiting the maximum transmission energy. Increasing roughness to 0.25 μm promotes irregular scattering, which reduces internal focusing and avoids severe internal damage, thereby increasing the maximum transmission energy but at the cost of lower transmission efficiency due to scattering losses. This trade-off highlights that roughness manipulation is beneficial for Gaussian lasers to prevent failure modes associated with high-energy-density spots.

In contrast, flat-top distributed lasers exhibit uniform energy distribution, which minimizes the risk of localized high-energy-density spot formation. With low roughness (0.08 μm), flat-top lasers achieve high transmission energy without significant internal damage, as the uniform energy profile reduces focusing effects. However, increasing roughness for flat-top lasers does not provide positive effects; instead, it enhances scattering, leading to reduced transmission efficiency and lower maximum energy. This indicates that flat-top lasers are inherently more suitable for high-energy transmission, and surface roughness optimization is unnecessary or even detrimental.

In summary, the study elucidates that flat-top laser sources are superior for high-energy applications due to their uniform distribution, while surface roughness control is primarily advantageous for Gaussian lasers to mitigate internal damage. These findings offer practical guidelines for designing laser fuzes to ensure efficient initiation of insensitive explosives, emphasizing the importance of matching energy distribution with fiber surface properties for optimal performance.

## Data Availability

The original contributions presented in this study are included in the article. Further inquiries can be directed to the corresponding authors.
